# [6-(Hy­droxy­meth­yl)pyridin-2-yl]methyl ferrocene-1-carboxyl­ate

**DOI:** 10.1107/S1600536811043972

**Published:** 2011-10-29

**Authors:** Mathieu Auzias, Georg Süss-Fink, Bruno Therrien

**Affiliations:** a Institut de Chimie, Université de Neuchâtel, Avenue de Bellevaux 51, CH-2000 Neuchâtel, Switzerland

## Abstract

The crystal structure of the title ferrocene derivative, [Fe(C_5_H_5_)(C_13_H_12_NO_3_)], shows strong inter­molecular O—H⋯N hydrogen bonds between the alcohol function and the pyridine group of a neighbouring mol­ecule, while the pyridine function forms another hydrogen bond with the alcohol function of another neighbouring mol­ecule, resulting in the formation of chains along the *a-*axis direction.

## Related literature

For analogous pyridino­ferrocene derivatives and the synthesis of the title compound, see: Izumi *et al.* (1984 ▶, 1988 ▶). For a review on ferrocene derivatives as anti­cancer agents, see: Hillard & Jaouen (2011 ▶). For related structures, see: Auzias *et al.* (2008 ▶, 2009 ▶).
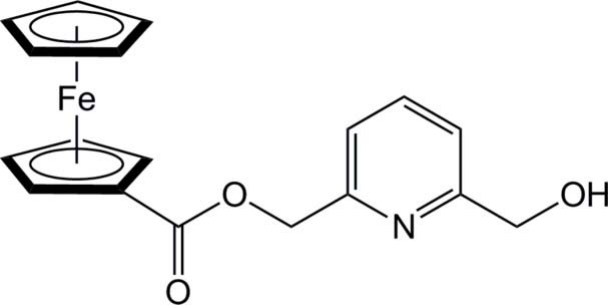


## Experimental

### 

#### Crystal data

[Fe(C_5_H_5_)(C_13_H_12_NO_3_)]
*M* *_r_* = 351.18
Tetragonal, 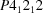

*a* = 7.5477 (3) Å
*c* = 53.351 (4) Å
*V* = 3039.3 (3) Å^3^
*Z* = 8
Mo *K*α radiation
μ = 1.01 mm^−1^
*T* = 173 K
0.17 × 0.14 × 0.12 mm

#### Data collection

Bruker SMART CCD diffractometer
13026 measured reflections
2808 independent reflections
2092 reflections with *I* > 2σ(*I*)
*R*_int_ = 0.072

#### Refinement

*R*[*F* ^2^ > 2σ(*F* ^2^)] = 0.038
*wR*(*F* ^2^) = 0.083
*S* = 0.86
2808 reflections
212 parameters
H atoms treated by a mixture of independent and constrained refinement
Δρ_max_ = 0.24 e Å^−3^
Δρ_min_ = −0.57 e Å^−3^
Absolute structure: Flack (1983 ▶), 1024 Friedel pairs
Flack parameter: 0.02 (3)

### 

Data collection: *SMART* (Bruker, 1999 ▶); cell refinement: *SMART* and *SAINT* (Bruker, 1999 ▶); data reduction: *SAINT*; program(s) used to solve structure: *SIR97* (Altomare *et al.*, 1999 ▶); program(s) used to refine structure: *SHELXTL* (Sheldrick, 2008 ▶); molecular graphics: *SHELXTL*; software used to prepare material for publication: *SHELXTL*.

## Supplementary Material

Crystal structure: contains datablock(s) I, global. DOI: 10.1107/S1600536811043972/ff2038sup1.cif

Structure factors: contains datablock(s) I. DOI: 10.1107/S1600536811043972/ff2038Isup2.hkl

Additional supplementary materials:  crystallographic information; 3D view; checkCIF report

## Figures and Tables

**Fig. 1.**
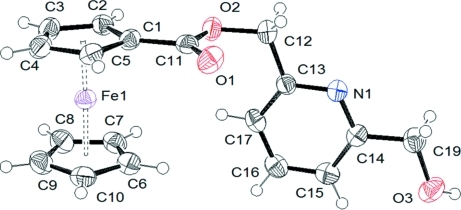
The molecular structure of {6-(hydroxymethyl)pyridin-2-yl}methyl ferrocene-1-carboxylate. Displacement ellipsoids are drawn at the 50% probability level.

## supplementary crystallographic information

### Comment

Ferrocene derivatives are known to exert antiproliferative effects on cancers (Hillard & Jaouen, 2011) and, in this paper, the molecular structure of a ferrocene derivative, {6-(hydroxymethyl)pyridin-2-yl}methyl ferrocene-1-carboxylate, is presented. However, a biological evaluation was not possible due to the low water-solubility of the compound.

The title compound is obtained following a previsouly published procedure (Izumi *et al.*, 1984; Izumi *et al.*, 1988) using ferrocenoyl chloride and 2,6-pyridinedimethanol in the presence of triethylamine. After purification, the compound crystallizes in the tetragonal space group *P* 4_1_2_1_2 with eight molecules per unit cell. These molecules are pilled along the *c* axis (*c* = 53.351 (4) Å). In the solid state, the ferrocene adopts an eclipsed conformation, and the pyridyl substituent is positionned perpendicular to the plane of the η^5^-C_5_H_4_COOCH_2_– moiety. Otherwise, the metrical parameters are normal and compare well to those of analogous functionalized-ferrocenyl molecules (Auzias *et al.*, 2008; Auzias *et al.*, 2009).

In the crystal packing, the pyridyl and the alcohol functions are involved in strong H-bonded interactions with two neighbouring symmetry-related molecules: The N—O separation being 2.827 (4) Å with the O—H···N angle being 159 (6)°.

### Experimental

Crystals suitable for X-ray diffraction analysis were obtained, after days, by slow diffusion of diethyl ether into a chloroform solution of the title complex.

### Refinement

All H atoms, except for OH, were included in calculated positions (C—H = 0.93 Å for C_arom_, 0.97 Å for CH_2_) and treated as riding atoms with the constraint *U*_iso_(H) = 1.2 *U*_eq_(carrier) applied.

### Crystal data

**Table:**

[Fe(C_5_H_5_)(C_13_H_12_NO_3_)]	*D*_x_ = 1.535 Mg m^−^^3^
*M**_r_* = 351.18	Mo *K*α radiation, λ = 0.71073 Å
Tetragonal, *P*4_1_2_1_2	Cell parameters from 9687 reflections
Hall symbol: P 4abw 2nw	θ = 1.5–26.0°
*a* = 7.5477 (3) Å	µ = 1.01 mm^−^^1^
*c* = 53.351 (4) Å	*T* = 173 K
*V* = 3039.3 (3) Å^3^	Block, orange
*Z* = 8	0.17 × 0.14 × 0.12 mm
*F*(000) = 1456	

### Data collection

**Table:**

Bruker SMART CCD diffractometer	2092 reflections with *I* > 2σ(*I*)
Radiation source: fine-focus sealed tube	*R*_int_ = 0.072
graphite	θ_max_ = 25.6°, θ_min_ = 1.5°
Detector resolution: 0 pixels mm^-1^	*h* = −9→8
ω scans	*k* = −9→9
13026 measured reflections	*l* = −64→64
2808 independent reflections	

### Refinement

**Table:**

Refinement on *F*^2^	Secondary atom site location: difference Fourier map
Least-squares matrix: full	Hydrogen site location: inferred from neighbouring sites
*R*[*F*^2^ > 2σ(*F*^2^)] = 0.038	H atoms treated by a mixture of independent and constrained refinement
*wR*(*F*^2^) = 0.083	*w* = 1/[σ^2^(*F*_o_^2^) + (0.0458*P*)^2^] where *P* = (*F*_o_^2^ + 2*F*_c_^2^)/3
*S* = 0.86	(Δ/σ)_max_ < 0.001
2808 reflections	Δρ_max_ = 0.24 e Å^−^^3^
212 parameters	Δρ_min_ = −0.57 e Å^−^^3^
0 restraints	Absolute structure: Flack (1983), 1024 Friedel pairs
Primary atom site location: structure-invariant direct methods	Flack parameter: 0.02 (3)

### Special details

**Table:**

Experimental. A crystal was mounted at 173 K on a Bruker *SMART* CCD PLATFORM using Mo *K*α graphite monochromated radiation. Image plate distance 70 mm, φ oscillation scans 0 - 200°, step Δφ = 0.5°, 3 minutes per frame.
Geometry. All e.s.d.'s (except the e.s.d. in the dihedral angle between two l.s. planes) are estimated using the full covariance matrix. The cell e.s.d.'s are taken into account individually in the estimation of e.s.d.'s in distances, angles and torsion angles; correlations between e.s.d.'s in cell parameters are only used when they are defined by crystal symmetry. An approximate (isotropic) treatment of cell e.s.d.'s is used for estimating e.s.d.'s involving l.s. planes.
Refinement. Refinement of *F*^2^ against ALL reflections. The weighted *R*-factor *wR* and goodness of fit *S* are based on *F*^2^, conventional *R*-factors *R* are based on *F*, with *F* set to zero for negative *F*^2^. The threshold expression of *F*^2^ > σ(*F*^2^) is used only for calculating *R*-factors(gt) *etc*. and is not relevant to the choice of reflections for refinement. *R*-factors based on *F*^2^ are statistically about twice as large as those based on *F*, and *R*- factors based on ALL data will be even larger.

### Fractional atomic coordinates and isotropic or equivalent isotropic displacement parameters (Å^2^)

**Table:**

	*x*	*y*	*z*	*U*_iso_*/*U*_eq_	
Fe1	0.16190 (7)	0.78987 (6)	0.032064 (10)	0.03038 (14)	
O2	−0.0794 (3)	0.8358 (3)	0.09355 (4)	0.0367 (6)	
O1	−0.2404 (3)	0.9840 (3)	0.06481 (6)	0.0436 (7)	
C11	−0.0980 (5)	0.9287 (5)	0.07208 (7)	0.0323 (8)	
C7	0.1223 (5)	0.5232 (5)	0.03604 (7)	0.0393 (9)	
H7	0.1192	0.4606	0.0510	0.047*	
C1	0.0706 (5)	0.9566 (4)	0.05913 (7)	0.0307 (8)	
C5	0.0887 (5)	1.0483 (5)	0.03606 (7)	0.0335 (8)	
H5	−0.0017	1.1044	0.0273	0.040*	
C9	0.2208 (5)	0.6541 (5)	−0.00017 (8)	0.0432 (10)	
H9	0.2945	0.6926	−0.0130	0.052*	
C4	0.2692 (5)	1.0391 (5)	0.02876 (8)	0.0375 (9)	
H4	0.3180	1.0894	0.0144	0.045*	
N1	−0.5056 (4)	0.6281 (4)	0.10764 (5)	0.0300 (7)	
C13	−0.3422 (4)	0.6468 (4)	0.09774 (6)	0.0304 (8)	
C14	−0.6050 (5)	0.4895 (5)	0.10050 (6)	0.0318 (9)	
C17	−0.2723 (5)	0.5255 (5)	0.08130 (7)	0.0365 (9)	
H17	−0.1574	0.5386	0.0753	0.044*	
C10	0.0354 (5)	0.6827 (5)	0.00140 (7)	0.0400 (9)	
H10	−0.0344	0.7431	−0.0101	0.048*	
C2	0.2408 (5)	0.8887 (5)	0.06595 (7)	0.0318 (8)	
H2	0.2673	0.8227	0.0802	0.038*	
C16	−0.3757 (5)	0.3827 (5)	0.07373 (7)	0.0381 (10)	
H16	−0.3321	0.3007	0.0623	0.046*	
C15	−0.5450 (5)	0.3646 (5)	0.08355 (7)	0.0361 (9)	
H15	−0.6167	0.2702	0.0788	0.043*	
C3	0.3618 (5)	0.9408 (5)	0.04698 (8)	0.0379 (9)	
H3	0.4821	0.9145	0.0466	0.046*	
C8	0.2748 (6)	0.5564 (5)	0.02136 (8)	0.0426 (10)	
H8	0.3897	0.5207	0.0251	0.051*	
C19	−0.7864 (5)	0.4814 (5)	0.11208 (8)	0.0399 (10)	
H19A	−0.7748	0.4622	0.1300	0.048*	
H19B	−0.8458	0.5939	0.1096	0.048*	
C6	−0.0237 (5)	0.6013 (5)	0.02401 (7)	0.0385 (9)	
H6	−0.1398	0.6001	0.0298	0.046*	
C12	−0.2403 (5)	0.8036 (5)	0.10734 (7)	0.0418 (9)	
H12A	−0.3148	0.9081	0.1064	0.050*	
H12B	−0.2110	0.7842	0.1248	0.050*	
O3	−0.8909 (4)	0.3441 (4)	0.10162 (6)	0.0475 (7)	
H3O	−0.935 (8)	0.248 (8)	0.1126 (13)	0.14 (3)*	

### Atomic displacement parameters (Å^2^)

**Table:**

	*U*^11^	*U*^22^	*U*^33^	*U*^12^	*U*^13^	*U*^23^
Fe1	0.0306 (3)	0.0287 (3)	0.0318 (2)	0.0000 (2)	−0.0007 (2)	−0.0016 (2)
O2	0.0287 (13)	0.0459 (16)	0.0356 (14)	−0.0041 (12)	0.0036 (11)	0.0020 (13)
O1	0.0284 (16)	0.0444 (16)	0.0581 (18)	0.0058 (12)	−0.0066 (13)	0.0012 (14)
C11	0.033 (2)	0.029 (2)	0.035 (2)	−0.0008 (16)	−0.0008 (16)	−0.0083 (16)
C7	0.050 (3)	0.034 (2)	0.034 (2)	0.0004 (17)	0.0023 (18)	−0.0036 (17)
C1	0.025 (2)	0.031 (2)	0.036 (2)	−0.0003 (14)	−0.0020 (15)	−0.0020 (16)
C5	0.034 (2)	0.031 (2)	0.035 (2)	−0.0041 (15)	−0.0046 (16)	0.0000 (16)
C9	0.051 (3)	0.038 (2)	0.040 (2)	−0.001 (2)	0.0090 (18)	−0.0070 (19)
C4	0.035 (2)	0.038 (2)	0.039 (2)	−0.0072 (16)	−0.0006 (17)	0.0016 (17)
N1	0.0288 (17)	0.0290 (18)	0.0323 (16)	0.0008 (13)	0.0012 (13)	0.0023 (13)
C13	0.0292 (19)	0.0329 (19)	0.0293 (17)	0.0014 (17)	0.0016 (14)	−0.0011 (14)
C14	0.032 (2)	0.031 (2)	0.033 (2)	−0.0004 (15)	−0.0016 (15)	0.0056 (16)
C17	0.036 (2)	0.039 (2)	0.034 (2)	0.0064 (17)	0.0060 (17)	−0.0020 (17)
C10	0.046 (3)	0.040 (2)	0.034 (2)	−0.0031 (18)	−0.0052 (17)	−0.0045 (19)
C2	0.032 (2)	0.035 (2)	0.0285 (18)	−0.0001 (15)	−0.0023 (15)	−0.0006 (15)
C16	0.047 (3)	0.030 (2)	0.038 (2)	0.0062 (17)	0.0044 (18)	0.0002 (16)
C15	0.047 (2)	0.028 (2)	0.033 (2)	−0.0003 (18)	−0.0017 (17)	0.0027 (16)
C3	0.028 (2)	0.041 (2)	0.045 (2)	−0.0030 (18)	0.0017 (17)	−0.0012 (17)
C8	0.044 (2)	0.042 (2)	0.041 (2)	0.0041 (19)	0.0026 (19)	−0.0038 (18)
C19	0.042 (2)	0.036 (2)	0.042 (2)	−0.0028 (18)	0.0048 (19)	0.0052 (17)
C6	0.041 (2)	0.037 (2)	0.037 (2)	0.0010 (17)	−0.0001 (18)	−0.0021 (17)
C12	0.036 (2)	0.051 (3)	0.039 (2)	−0.0074 (17)	0.0099 (16)	−0.0056 (19)
O3	0.0446 (17)	0.0500 (18)	0.0478 (17)	−0.0159 (14)	−0.0041 (13)	0.0078 (15)

### Geometric parameters (Å, °)

**Table:**

Fe1—C1	2.035 (3)	C4—H4	0.9300
Fe1—C5	2.038 (4)	N1—C14	1.342 (4)
Fe1—C8	2.039 (4)	N1—C13	1.349 (4)
Fe1—C6	2.042 (4)	C13—C17	1.373 (5)
Fe1—C2	2.045 (3)	C13—C12	1.502 (5)
Fe1—C7	2.046 (4)	C14—C15	1.382 (5)
Fe1—C9	2.051 (4)	C14—C19	1.504 (5)
Fe1—C3	2.051 (4)	C17—C16	1.390 (5)
Fe1—C4	2.056 (3)	C17—H17	0.9300
Fe1—C10	2.060 (4)	C10—C6	1.425 (5)
O2—C11	1.350 (4)	C10—H10	0.9300
O2—C12	1.440 (4)	C2—C3	1.419 (5)
O1—C11	1.217 (4)	C2—H2	0.9300
C11—C1	1.464 (5)	C16—C15	1.388 (5)
C7—C6	1.404 (5)	C16—H16	0.9300
C7—C8	1.414 (5)	C15—H15	0.9300
C7—H7	0.9300	C3—H3	0.9300
C1—C5	1.419 (5)	C8—H8	0.9300
C1—C2	1.430 (5)	C19—O3	1.416 (4)
C5—C4	1.418 (5)	C19—H19A	0.9700
C5—H5	0.9300	C19—H19B	0.9700
C9—C10	1.418 (5)	C6—H6	0.9300
C9—C8	1.425 (6)	C12—H12A	0.9700
C9—H9	0.9300	C12—H12B	0.9700
C4—C3	1.408 (5)	O3—H3O	0.99 (6)
			
C1—Fe1—C5	40.77 (13)	C8—C9—Fe1	69.2 (2)
C1—Fe1—C8	150.96 (15)	C10—C9—H9	125.9
C5—Fe1—C8	165.83 (15)	C8—C9—H9	125.9
C1—Fe1—C6	110.37 (15)	Fe1—C9—H9	126.3
C5—Fe1—C6	120.19 (15)	C3—C4—C5	108.2 (4)
C8—Fe1—C6	67.99 (16)	C3—C4—Fe1	69.8 (2)
C1—Fe1—C2	41.02 (13)	C5—C4—Fe1	69.07 (19)
C5—Fe1—C2	68.73 (14)	C3—C4—H4	125.9
C8—Fe1—C2	116.19 (16)	C5—C4—H4	125.9
C6—Fe1—C2	129.80 (14)	Fe1—C4—H4	126.9
C1—Fe1—C7	119.02 (14)	C14—N1—C13	118.7 (3)
C5—Fe1—C7	152.99 (14)	N1—C13—C17	122.1 (3)
C8—Fe1—C7	40.51 (14)	N1—C13—C12	114.6 (3)
C6—Fe1—C7	40.19 (15)	C17—C13—C12	123.1 (3)
C2—Fe1—C7	108.06 (15)	N1—C14—C15	122.3 (3)
C1—Fe1—C9	167.76 (15)	N1—C14—C19	115.1 (3)
C5—Fe1—C9	128.65 (16)	C15—C14—C19	122.6 (3)
C8—Fe1—C9	40.78 (15)	C13—C17—C16	119.1 (4)
C6—Fe1—C9	67.92 (15)	C13—C17—H17	120.4
C2—Fe1—C9	149.40 (15)	C16—C17—H17	120.4
C7—Fe1—C9	68.11 (15)	C9—C10—C6	107.1 (4)
C1—Fe1—C3	68.30 (15)	C9—C10—Fe1	69.5 (2)
C5—Fe1—C3	68.11 (14)	C6—C10—Fe1	69.0 (2)
C8—Fe1—C3	106.35 (16)	C9—C10—H10	126.5
C6—Fe1—C3	166.67 (15)	C6—C10—H10	126.5
C2—Fe1—C3	40.54 (14)	Fe1—C10—H10	126.6
C7—Fe1—C3	127.87 (15)	C3—C2—C1	107.3 (3)
C9—Fe1—C3	116.32 (15)	C3—C2—Fe1	70.0 (2)
C1—Fe1—C4	68.18 (15)	C1—C2—Fe1	69.1 (2)
C5—Fe1—C4	40.54 (14)	C3—C2—H2	126.4
C8—Fe1—C4	127.05 (15)	C1—C2—H2	126.4
C6—Fe1—C4	152.84 (16)	Fe1—C2—H2	126.1
C2—Fe1—C4	68.10 (15)	C15—C16—C17	118.9 (4)
C7—Fe1—C4	165.19 (15)	C15—C16—H16	120.5
C9—Fe1—C4	107.45 (16)	C17—C16—H16	120.5
C3—Fe1—C4	40.10 (15)	C14—C15—C16	118.8 (3)
C1—Fe1—C10	130.51 (16)	C14—C15—H15	120.6
C5—Fe1—C10	109.47 (16)	C16—C15—H15	120.6
C8—Fe1—C10	68.39 (17)	C4—C3—C2	108.6 (3)
C6—Fe1—C10	40.65 (14)	C4—C3—Fe1	70.1 (2)
C2—Fe1—C10	168.74 (14)	C2—C3—Fe1	69.5 (2)
C7—Fe1—C10	68.17 (16)	C4—C3—H3	125.7
C9—Fe1—C10	40.37 (15)	C2—C3—H3	125.7
C3—Fe1—C10	150.13 (16)	Fe1—C3—H3	126.3
C4—Fe1—C10	118.30 (16)	C7—C8—C9	107.8 (4)
C11—O2—C12	115.7 (3)	C7—C8—Fe1	70.0 (2)
O1—C11—O2	122.7 (3)	C9—C8—Fe1	70.0 (2)
O1—C11—C1	124.6 (4)	C7—C8—H8	126.1
O2—C11—C1	112.6 (3)	C9—C8—H8	126.1
C6—C7—C8	108.1 (3)	Fe1—C8—H8	125.4
C6—C7—Fe1	69.8 (2)	O3—C19—C14	112.0 (3)
C8—C7—Fe1	69.5 (2)	O3—C19—H19A	109.2
C6—C7—H7	125.9	C14—C19—H19A	109.2
C8—C7—H7	125.9	O3—C19—H19B	109.2
Fe1—C7—H7	126.4	C14—C19—H19B	109.2
C5—C1—C2	108.0 (3)	H19A—C19—H19B	107.9
C5—C1—C11	124.3 (3)	C7—C6—C10	108.8 (4)
C2—C1—C11	127.6 (3)	C7—C6—Fe1	70.0 (2)
C5—C1—Fe1	69.7 (2)	C10—C6—Fe1	70.3 (2)
C2—C1—Fe1	69.8 (2)	C7—C6—H6	125.6
C11—C1—Fe1	122.7 (2)	C10—C6—H6	125.6
C4—C5—C1	107.9 (3)	Fe1—C6—H6	125.6
C4—C5—Fe1	70.4 (2)	O2—C12—C13	113.0 (3)
C1—C5—Fe1	69.5 (2)	O2—C12—H12A	109.0
C4—C5—H5	126.1	C13—C12—H12A	109.0
C1—C5—H5	126.1	O2—C12—H12B	109.0
Fe1—C5—H5	125.6	C13—C12—H12B	109.0
C10—C9—C8	108.2 (4)	H12A—C12—H12B	107.8
C10—C9—Fe1	70.2 (2)	C19—O3—H3O	119 (4)
			
C12—O2—C11—O1	0.8 (5)	C8—C9—C10—Fe1	58.9 (3)
C12—O2—C11—C1	179.9 (3)	C1—Fe1—C10—C9	168.4 (2)
C1—Fe1—C7—C6	87.7 (2)	C5—Fe1—C10—C9	127.4 (2)
C5—Fe1—C7—C6	52.7 (4)	C8—Fe1—C10—C9	−37.7 (2)
C8—Fe1—C7—C6	−119.4 (3)	C6—Fe1—C10—C9	−118.6 (4)
C2—Fe1—C7—C6	131.1 (2)	C2—Fe1—C10—C9	−153.9 (7)
C9—Fe1—C7—C6	−81.3 (2)	C7—Fe1—C10—C9	−81.4 (3)
C3—Fe1—C7—C6	171.7 (2)	C3—Fe1—C10—C9	47.6 (4)
C4—Fe1—C7—C6	−156.5 (6)	C4—Fe1—C10—C9	83.8 (3)
C10—Fe1—C7—C6	−37.6 (2)	C1—Fe1—C10—C6	−73.0 (3)
C1—Fe1—C7—C8	−152.9 (2)	C5—Fe1—C10—C6	−114.0 (2)
C5—Fe1—C7—C8	172.1 (3)	C8—Fe1—C10—C6	80.9 (3)
C6—Fe1—C7—C8	119.4 (3)	C2—Fe1—C10—C6	−35.2 (9)
C2—Fe1—C7—C8	−109.5 (2)	C7—Fe1—C10—C6	37.2 (2)
C9—Fe1—C7—C8	38.2 (2)	C9—Fe1—C10—C6	118.6 (4)
C3—Fe1—C7—C8	−68.8 (3)	C3—Fe1—C10—C6	166.3 (3)
C4—Fe1—C7—C8	−37.0 (7)	C4—Fe1—C10—C6	−157.5 (2)
C10—Fe1—C7—C8	81.8 (3)	C5—C1—C2—C3	0.3 (4)
O1—C11—C1—C5	−1.6 (6)	C11—C1—C2—C3	176.2 (3)
O2—C11—C1—C5	179.3 (3)	Fe1—C1—C2—C3	59.8 (2)
O1—C11—C1—C2	−176.8 (4)	C5—C1—C2—Fe1	−59.5 (2)
O2—C11—C1—C2	4.1 (5)	C11—C1—C2—Fe1	116.3 (3)
O1—C11—C1—Fe1	−88.2 (4)	C1—Fe1—C2—C3	−118.5 (3)
O2—C11—C1—Fe1	92.8 (3)	C5—Fe1—C2—C3	−80.8 (2)
C8—Fe1—C1—C5	165.9 (3)	C8—Fe1—C2—C3	84.7 (3)
C6—Fe1—C1—C5	−113.0 (2)	C6—Fe1—C2—C3	167.0 (2)
C2—Fe1—C1—C5	119.1 (3)	C7—Fe1—C2—C3	127.7 (2)
C7—Fe1—C1—C5	−156.5 (2)	C9—Fe1—C2—C3	50.4 (4)
C9—Fe1—C1—C5	−33.4 (8)	C4—Fe1—C2—C3	−37.0 (2)
C3—Fe1—C1—C5	81.2 (2)	C10—Fe1—C2—C3	−163.7 (7)
C4—Fe1—C1—C5	37.9 (2)	C5—Fe1—C2—C1	37.74 (19)
C10—Fe1—C1—C5	−71.4 (3)	C8—Fe1—C2—C1	−156.8 (2)
C5—Fe1—C1—C2	−119.1 (3)	C6—Fe1—C2—C1	−74.5 (3)
C8—Fe1—C1—C2	46.8 (4)	C7—Fe1—C2—C1	−113.7 (2)
C6—Fe1—C1—C2	127.9 (2)	C9—Fe1—C2—C1	168.9 (3)
C7—Fe1—C1—C2	84.4 (2)	C3—Fe1—C2—C1	118.5 (3)
C9—Fe1—C1—C2	−152.5 (7)	C4—Fe1—C2—C1	81.5 (2)
C3—Fe1—C1—C2	−37.9 (2)	C10—Fe1—C2—C1	−45.2 (9)
C4—Fe1—C1—C2	−81.3 (2)	C13—C17—C16—C15	−1.7 (6)
C10—Fe1—C1—C2	169.5 (2)	N1—C14—C15—C16	0.4 (5)
C5—Fe1—C1—C11	118.5 (4)	C19—C14—C15—C16	179.8 (3)
C8—Fe1—C1—C11	−75.6 (4)	C17—C16—C15—C14	0.1 (6)
C6—Fe1—C1—C11	5.5 (3)	C5—C4—C3—C2	−0.5 (4)
C2—Fe1—C1—C11	−122.4 (4)	Fe1—C4—C3—C2	−59.0 (3)
C7—Fe1—C1—C11	−38.0 (4)	C5—C4—C3—Fe1	58.5 (2)
C9—Fe1—C1—C11	85.1 (8)	C1—C2—C3—C4	0.1 (4)
C3—Fe1—C1—C11	−160.3 (3)	Fe1—C2—C3—C4	59.4 (3)
C4—Fe1—C1—C11	156.3 (3)	C1—C2—C3—Fe1	−59.3 (2)
C10—Fe1—C1—C11	47.1 (4)	C1—Fe1—C3—C4	−81.5 (2)
C2—C1—C5—C4	−0.6 (4)	C5—Fe1—C3—C4	−37.4 (2)
C11—C1—C5—C4	−176.7 (3)	C8—Fe1—C3—C4	128.8 (2)
Fe1—C1—C5—C4	−60.2 (3)	C6—Fe1—C3—C4	−168.3 (6)
C2—C1—C5—Fe1	59.6 (2)	C2—Fe1—C3—C4	−119.8 (3)
C11—C1—C5—Fe1	−116.4 (3)	C7—Fe1—C3—C4	167.9 (2)
C1—Fe1—C5—C4	118.7 (3)	C9—Fe1—C3—C4	86.1 (3)
C8—Fe1—C5—C4	−32.4 (7)	C10—Fe1—C3—C4	53.8 (4)
C6—Fe1—C5—C4	−154.6 (2)	C1—Fe1—C3—C2	38.4 (2)
C2—Fe1—C5—C4	80.8 (2)	C5—Fe1—C3—C2	82.4 (2)
C7—Fe1—C5—C4	169.0 (3)	C8—Fe1—C3—C2	−111.4 (2)
C9—Fe1—C5—C4	−69.9 (3)	C6—Fe1—C3—C2	−48.5 (7)
C3—Fe1—C5—C4	37.0 (2)	C7—Fe1—C3—C2	−72.2 (3)
C10—Fe1—C5—C4	−111.1 (2)	C9—Fe1—C3—C2	−154.1 (2)
C8—Fe1—C5—C1	−151.1 (6)	C4—Fe1—C3—C2	119.8 (3)
C6—Fe1—C5—C1	86.7 (2)	C10—Fe1—C3—C2	173.7 (3)
C2—Fe1—C5—C1	−38.0 (2)	C6—C7—C8—C9	−0.8 (4)
C7—Fe1—C5—C1	50.2 (4)	Fe1—C7—C8—C9	−60.1 (3)
C9—Fe1—C5—C1	171.4 (2)	C6—C7—C8—Fe1	59.3 (3)
C3—Fe1—C5—C1	−81.7 (2)	C10—C9—C8—C7	0.6 (4)
C4—Fe1—C5—C1	−118.7 (3)	Fe1—C9—C8—C7	60.1 (3)
C10—Fe1—C5—C1	130.2 (2)	C10—C9—C8—Fe1	−59.5 (3)
C1—Fe1—C9—C10	−46.3 (8)	C1—Fe1—C8—C7	55.3 (4)
C5—Fe1—C9—C10	−73.6 (3)	C5—Fe1—C8—C7	−165.3 (6)
C8—Fe1—C9—C10	119.5 (3)	C6—Fe1—C8—C7	−37.3 (2)
C6—Fe1—C9—C10	38.1 (2)	C2—Fe1—C8—C7	87.5 (2)
C2—Fe1—C9—C10	170.3 (3)	C9—Fe1—C8—C7	−118.6 (3)
C7—Fe1—C9—C10	81.6 (2)	C3—Fe1—C8—C7	129.9 (2)
C3—Fe1—C9—C10	−155.8 (2)	C4—Fe1—C8—C7	168.9 (2)
C4—Fe1—C9—C10	−113.4 (2)	C10—Fe1—C8—C7	−81.3 (2)
C1—Fe1—C9—C8	−165.8 (6)	C1—Fe1—C8—C9	173.8 (3)
C5—Fe1—C9—C8	166.8 (2)	C5—Fe1—C8—C9	−46.7 (7)
C6—Fe1—C9—C8	−81.4 (2)	C6—Fe1—C8—C9	81.3 (2)
C2—Fe1—C9—C8	50.7 (4)	C2—Fe1—C8—C9	−154.0 (2)
C7—Fe1—C9—C8	−37.9 (2)	C7—Fe1—C8—C9	118.6 (3)
C3—Fe1—C9—C8	84.7 (3)	C3—Fe1—C8—C9	−111.5 (2)
C4—Fe1—C9—C8	127.0 (2)	C4—Fe1—C8—C9	−72.6 (3)
C10—Fe1—C9—C8	−119.5 (3)	C10—Fe1—C8—C9	37.3 (2)
C1—C5—C4—C3	0.7 (4)	N1—C14—C19—O3	173.3 (3)
Fe1—C5—C4—C3	−58.9 (3)	C15—C14—C19—O3	−6.2 (5)
C1—C5—C4—Fe1	59.7 (2)	C8—C7—C6—C10	0.7 (4)
C1—Fe1—C4—C3	81.8 (3)	Fe1—C7—C6—C10	59.9 (3)
C5—Fe1—C4—C3	119.9 (3)	C8—C7—C6—Fe1	−59.1 (3)
C8—Fe1—C4—C3	−69.6 (3)	C9—C10—C6—C7	−0.4 (4)
C6—Fe1—C4—C3	174.1 (3)	Fe1—C10—C6—C7	−59.7 (3)
C2—Fe1—C4—C3	37.4 (2)	C9—C10—C6—Fe1	59.3 (3)
C7—Fe1—C4—C3	−40.3 (7)	C1—Fe1—C6—C7	−111.3 (2)
C9—Fe1—C4—C3	−110.4 (2)	C5—Fe1—C6—C7	−155.3 (2)
C10—Fe1—C4—C3	−152.8 (2)	C8—Fe1—C6—C7	37.6 (2)
C1—Fe1—C4—C5	−38.1 (2)	C2—Fe1—C6—C7	−68.8 (3)
C8—Fe1—C4—C5	170.5 (2)	C9—Fe1—C6—C7	81.8 (2)
C6—Fe1—C4—C5	54.3 (5)	C3—Fe1—C6—C7	−29.5 (8)
C2—Fe1—C4—C5	−82.4 (2)	C4—Fe1—C6—C7	167.1 (3)
C7—Fe1—C4—C5	−160.1 (5)	C10—Fe1—C6—C7	119.6 (3)
C9—Fe1—C4—C5	129.8 (2)	C1—Fe1—C6—C10	129.1 (2)
C3—Fe1—C4—C5	−119.9 (3)	C5—Fe1—C6—C10	85.1 (3)
C10—Fe1—C4—C5	87.3 (3)	C8—Fe1—C6—C10	−82.0 (3)
C14—N1—C13—C17	−2.3 (5)	C2—Fe1—C6—C10	171.6 (2)
C14—N1—C13—C12	−178.8 (3)	C7—Fe1—C6—C10	−119.6 (3)
C13—N1—C14—C15	0.6 (5)	C9—Fe1—C6—C10	−37.8 (2)
C13—N1—C14—C19	−178.8 (3)	C3—Fe1—C6—C10	−149.1 (6)
N1—C13—C17—C16	2.8 (5)	C4—Fe1—C6—C10	47.5 (5)
C12—C13—C17—C16	179.0 (4)	C11—O2—C12—C13	82.9 (4)
C8—C9—C10—C6	−0.1 (4)	N1—C13—C12—O2	−170.6 (3)
Fe1—C9—C10—C6	−59.0 (3)	C17—C13—C12—O2	13.0 (5)

### Hydrogen-bond geometry (Å, °)

**Table:**

*D*—H···*A*	*D*—H	H···*A*	*D*···*A*	*D*—H···*A*
O3—H3o···N1^i^	0.99 (6)	1.88 (7)	2.827 (4)	159 (6)

Symmetry codes: (i) −*x*−3/2, *y*−1/2, −*z*+1/4.
